# Supplementary material for: Large-Scale Seedling Grow-Out Experiments Do Not Support Seed Transmission of Sweet Potato Leaf Curl Virus in Sweet Potato
Source: Plants (Basel). 2021 Jan 12;10(1):139. doi: 10.3390/plants10010139 (PMC7827154; doi:10.3390/plants10010139)
Supplement: Supplementary file 1 [file plants-10-00139-s001.pdf]

Large Scale Seedling Grow-out Experiments Do Not Support Seed Transmission of Sweet Potato Leaf Curl Virus in Sweetpotato

Sharon A. Andreason, Omotola G. Olaniyi, Andrea C. Gilliard, Phillip A. Wadl, Livy H. Williams, D. Michael Jackson, Alvin M. Simmons, and Kai-Shu Ling

Supplementary files:

Supplementary Table S1. Primers used for sweet potato leaf curl virus (SPLCV) detection by real-time polymerase chain rection (qPCR) or end-point PCR.

**Primer Designation**	**Primer Name**	**Sequence 5'-3'**	**Reference**
**Real-time PCR for SPLCV (68 bp)**			
KL07-40	SPLCV.F2	5'GAG ACA GCT ATC GTG CC	[17]
KL07-41	SPLCV.R2	5'GAA ACC GGG ACA TAG CTT CG	
KL07-42	SPLCV.P2	5'FAM-TAC ACT GGG AAT GCT GTC CCA ATT GCT-TAMRA	
**Real-time PCR for 18S rRNA for an Internal Control**			
KL13-53	18S rRNA 449_F	5′ GTGACGGAGAATTAGGGTTCGA 3′	[45]
KL13-54	18S rRNA 498_R	5′ CTGCCTTCCTTGGATGTGGTA 3′	
KL13-55	18S rRNA 475_P	CY5-CCGGAGAGGGAGCCTGAGAAACGG-BHQ-2	
**Conventional End Point PCR for SPLCV Coat Protein Gene**			
KL09-14	SPLCV CP-F	5'-AAG AAA TAC GAG CCA GGA AC	This study
KL09-15	SPLCV CP-R1	5' TAT TAA TTA TTG TGC GAA TCA	

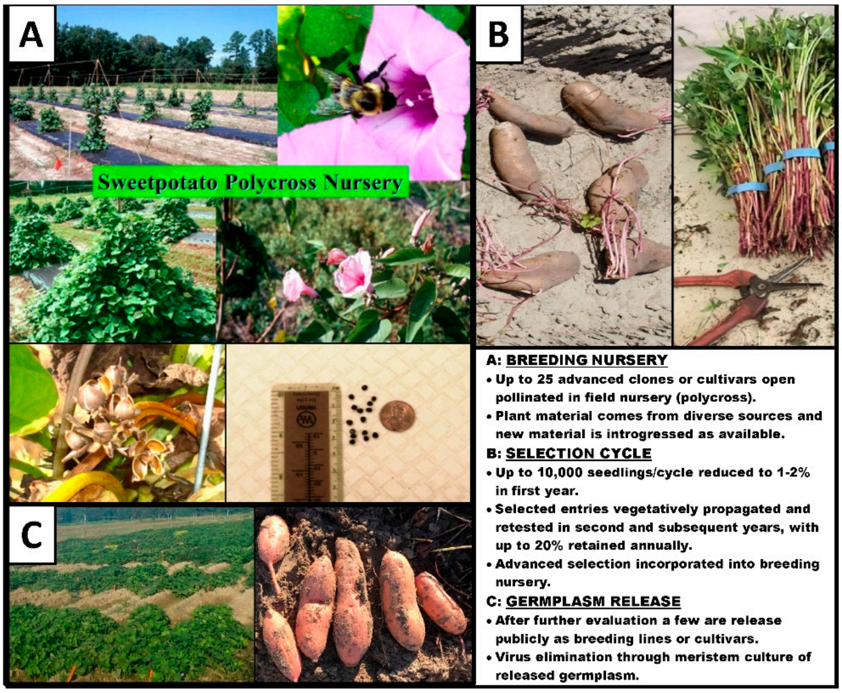

Supplementary Figure S1. Schematic of the current workflow in the USDA, ARS, U.S. Vegetable Laboratory Sweetpotato Breeding Program. The activities are recurring annually, with potential virus acquisition occurring during each cycle of selection. In the breeding nursery parental entries are open pollinated and seed is harvested for selection of improved germplasm. The selected germplasm is stored overwinter and planted in the spring for slip production. Slips are planted into replicated field plots and only a tiny fraction are ever released as breeding lines or cultivars.

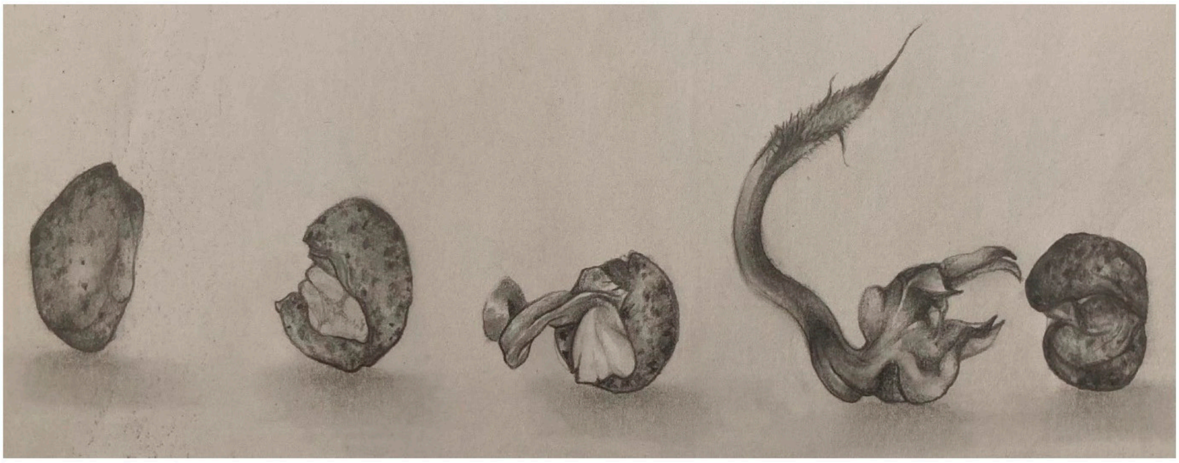

Supplementary Figure S2. Botanical drawing of sweetpotato seed germination over seven-day period for seeds germinated on sterilized moistened filter paper in glass Petri dishes for testing the emerging young seedling and seed coat for sweet potato leaf curl virus (SPLCV). From left to right: seed imbibing, seed coat cracks with visible embryo, radicle emergence, and seedling with cotyledons dehisces seed coat. The seedling and seed coat were then tested for SPLCV using real-time PCR.
